# Cost of illness of breast cancer in Japan: trends and future projections

**DOI:** 10.1186/s13104-015-1516-y

**Published:** 2015-10-05

**Authors:** Kunichika Matsumoto, Kayoko Haga, Takefumi Kitazawa, Kanako Seto, Shigeru Fujita, Tomonori Hasegawa

**Affiliations:** Department of Social Medicine, Toho University School of Medicine, 5-21-16 Omori-nishi, Ota-ku, Tokyo, 143-8540 Japan

**Keywords:** Cost of illness, Breast cancer, Health economics, Health policy

## Abstract

**Background:**

Breast cancer is a major cause of death for women in Japan. The objectives of this study were to estimate and project the economic burden associated with breast cancer in Japan and identify the key factors that drive the change of the economic burden of breast cancer.

**Methods:**

We calculated the cost of illness (COI) every 3 years from 1996 to 2020 using governmental statistics. COI was calculated by summing the direct costs, morbidity costs, and mortality costs.

**Results:**

From 1996 to 2011 COI was trending upward. COI in 2011 (697 billion yen) was 1.7-times greater than that in 1996 (407 billion yen). The mortality costs accounted for approximately 65–70 % of the total COI and were a major contributing factor to increase in COI. It was predicted that COI would continue to trend upwards until 2020 (699.4–743.8 billion yen depending on the model), but the rate of increase would decline.

**Conclusions:**

COI of breast cancer has been steadily increasing since 1996. While the rate of increase is expected to plateau, the average age at death from breast cancer is still less than that from other cancers, and the relative economic burden of breast cancer will continue to increase in the foreseeable future.

## Background

Breast cancer (ICD 10 code: C50) is the most prevalent cancer among women and the fifth leading cause of death [1]. In the past, the prevalence and mortality rate of breast cancer has been lower in Japan than in the United States and Europe [2–4], but have been increasing rapidly [5–8]. The salient feature of breast cancer in Japanese women is that the peak incidence is in women in their late forties, whereas in the United States and Europe the peak incidence is in women over 60 years of age [9–11]. For this reason, breast cancer causes severe damage for Japanese women in the prime of their life, resulting in a high economic burden of treatment for breast cancer in Japan.

To date, only a few studies have attempted to estimate the economic burden of breast cancer in Japan [12, 13]. Moreover, most of them are limited to the estimation of direct medical expenses at a single time point. It is difficult to estimate the real social burden of a disease like breast cancer, where the incidence and mortality are high in younger women, using only the direct medical expenses.

In this study, we calculated direct costs as well as indirect costs, which include the opportunity cost because of disease and death. The goal was to adequately capture the social burden of breast cancer by estimating past trends and projecting future trends in the costs of breast cancer. We already tried to calculate COI of several cancers in the past [14]. But this study performed more detailed analysis of breast cancer and tried to project COI in the near future.

A previous study calculated the cost of illness (COI) of stomach cancer in Japan [15]. The COI calculations published for 1996, 2002, 2008, 2014, and 2020 concluded that COI decreased continuously until 2008. This is likely because of the devaluation of human capital with aging, particularly approaching the average age of death. The study further concluded that COI would continue to decrease in the near future.

Comparing with stomach cancer, the social burden of breast cancer is expected to increase. We compare our COI estimates for breast cancer with those for stomach cancer to show an evidence for prioritizing policies for cancer control.

## Methods

### Time-series estimation of COI

The COI method is well described for measuring the social burden of disease [16–22]. In this study, COI was calculated from 1996 to 2011 and, based on these data, future projections were made for 2014–2020 to evaluate trends over time.

The COI calculation is dependent on three variables: direct costs, morbidity costs, and mortality costs. The direct costs are defined as medical expenses (treatment costs, hospital charges, laboratory costs, drug costs, etc.). In this study, we used reimbursement data from the “Survey of National Medical Care Insurance Services” to calculate annual medical expenses.

The morbidity costs are associated with inpatient care and outpatient care. The morbidity costs of inpatients were calculated by multiplying total person-days of hospitalization by the 1-day labor-value per person. The morbidity costs of outpatients were calculated by multiplying total person-days of outpatient visits by half the 1-day labor-value per person. Total person-days of outpatient visits and hospitalization according to sex and 5 years age-groups were calculated based on the “Patient Survey”. The labor-value was calculated according to sex and 5 years age-groups using the “Basic Survey on Wage Structure”, “Labor Force Survey”, and “Estimates of monetary valuation of unpaid work”.

The mortality costs are measured as the loss of human capital (human capital method), which was calculated by multiplying the number of deaths by the lifetime labor-value per person. The number of deaths caused by breast cancer according to sex and 5 years age-groups was obtained from “Vital Statistics”. The lifetime labor-value was calculated by summing the income, which the person could have earned in the future if they had not died, from the year of death to life expectancy. The future labor-value was adjusted to a present value using a 3 % discount rate.

### Future projection of COI

Predictions of future COI from 2014 to 2020 were based on the “Population Projection for Japan: 2011–2060 (January 2012)” by the National Institute of Population and Social Security Research. The year 2011 was selected as the benchmark for the 1-day labor-value by sex and 5 years age-groups. Two methods were utilized for the future projection of COI. The first is the “fixed” method, which fixes health-related indicators (the mortality rate, number of times of outpatient visit per population, number of times of hospitalization per population, and average length of stay) of each age-group at the 2011 level and changes future population and age structure. The other is the “variable” method, which estimates health-related indicators in addition to population and age structure. Future health-related indicators are estimated using linear regression (linear model), logarithmic regression (logarithmic model), or a combination of regressions of higher coefficient of determination (mixed model). The details of the methodology are outlined in the COI study of stomach cancer [15]. The present study found that the mixed model was the most valid.

This study used only aggregated data, and did not use human or animals. In Japan, for this kind of study no institutional review is requested [23].

## Results

### COI from 1996 to 2011

Table 1 shows the trend of COI and health-related indicators from 1996 to 2011. COI was calculated to be 697 billion yen (≒6.97 billion dollar) in 2011. The contribution of the direct costs, morbidity costs, and mortality costs were 116.3 billion yen, 46.8 billion yen, and 484.0 billion yen, respectively. The mortality costs were the greatest contributors and accounted for 69.4 % of the total COI. COI increased continuously from 1996 to 2011 by 3.6 % annually for a total increase of 1.7-times. The direct costs increased until 2005 and decreased gradually after. The morbidity costs were almost constant, but the mortality costs, which accounted for approximately 70 % of COI, increased consistently. The contribution ratio of the mortality cost to total increase was 62.8 %.

**Table 1:** Trend of the cost of illness (COI) of breast cancer

	1996	1999	2002	2005	2008	2011
Population (thousand person)	125,864	126,686	127,435	127,768	127,692	127,799
[% of 65 years or older]	15.1 %	16.7 %	18.5 %	20.2 %	22.1 %	23.1 %
Number of breast cancer deaths (person)	7,900	8,882	9,604	10,721	11,797	12,731
[% of 65 years or older]	36.3 %	37.7 %	40.5 %	43.7 %	48.6 %	52.0 %
Average age of death (years)	60.1	61.0	62.1	63.3	64.8	66.1
Crude incidence rate (per 100 thousand, female)	47.9	54.4	64.4	77.5	99.5	NA
Crude mortality rate (per 100 thousand, female)	12.4	13.9	14.9	16.6	18.3	19.7
Fatality rate (female)	0.26	0.25	0.23	0.21	0.18	NA
Direct cost (billion yen)	72.2	124.9	146.8	185.4	168.6	166.3
Morbidity cost (billion yen)	33.3	40.1	49.0	43.7	47.2	46.8
Mortality cost (billion yen)	302.8	328.4	395.9	415.7	435.6	484.0
[% of 65 years or older]	11.2 %	11.5 %	14.8 %	16.2 %	19.3 %	23.1 %
Mortality cost per person (million yen)	38.3	37.0	41.2	38.8	36.9	38.0
COI (billion yen)	408.4	493.3	591.6	644.8	651.3	697.0

Source of population: Ministry of Internal Affairs and Communications “Population Estimates”. Source of the number of breast cancer deaths: “Vital Statistics”. Average age of death: calculated according to the number of deaths, sex and age (5 years old age grade), cause of death in “Vital Statistics”. Source of crude morbidity rate and crude mortality rate: Center for Cancer Control and Information Services, National Cancer Center, Japan. Fatality rate: we calculated by dividing the crude mortality rate by crude morbidity rate

*NA* not available

Increased mortality costs were the primary factor contributing to increased COI. Because the mortality cost per person (mortality cost/the number of deaths) was stable (36.9–41.2 million yen), it is likely that the increase in the number of deaths directly led to increases in the mortality costs. According to the National Cancer Center of Japan, the number of deaths increased 61.2 % from 1996 to 2011 [1]. The increase was continuous with an annual percent change (APC) of 3.2 %. The crude mortality rate showed a 58.3 % increase from 12.4 (per 100 thousand persons) in 1996 to 19.7 (per 100 thousand persons) in 2011. The crude incidence rate increased 2-times from 47.9 (per 100 thousand persons) in 1996 to 99.5 (per 100 thousand persons) in 2008, while the fatality rate decreased.

Table 2 shows the comparison of the mortality rate, number of deaths, and incidence rate according to sex and 5 years age-groups between 1996 and 2011. The mortality rate showed almost no increase in persons younger than 55 years, but there was a large increase (>30 % increase) in persons aged 55 years and older. Moreover, aging of the population accelerated the increase in the mortality rate in older persons. As a result, the number of deaths, average age at death, and associated mortality costs increased for persons aged 65 years or older (Table 1). Finally, the rate of increase exceeded 100 % in the 75–79 age-group.

**Table 2:** Mortality rate and the number of deaths due to breast cancer (1996 and 2011)

	25–29	30–34	35–39	40–44	45–49	50–54	55–59	60–64	65–69	70–74	75–79	80–84	85
Crude mortality rate (per 100 thousand, female)													
1996	0.6	2.2	7.0	11.7	18.8	26.3	25.2	24.6	25.2	24.2	24.2	25.2	34.3
2011	0.5	1.9	4.9	10.2	18.9	27.7	37.2	37.1	37.0	32.4	33.2	41.0	53.7
Changing rate	−22.4 %	−11.9 %	−30.5 %	−12.6 %	0.6 %	5.1 %	47.5 %	51.1 %	46.5 %	34.1 %	37.2 %	62.6 %	56.8 %
Number of breast cancer deaths (person)													
1996	27	85	265	494	1042	1120	1036	963	873	692	507	385	408
2011	16	75	228	461	735	1043	1545	2000	1509	1241	1146	1117	1567
Changing rate	−40.7 %	−11.8 %	−14.0 %	−6.7 %	−29.5 %	−6.9 %	49.1 %	107.7 %	72.9 %	79.3 %	126.0 %	190.1 %	284.1 %
Crude incidence rate (per 100 thousand, female)													
1996	4.3	12.8	38.2	71.5	115.4	89.5	83.1	80.4	81.9	85.1	73.9	66.6	59.8
2008	8.1	28.1	57.5	130.9	195.7	179.6	177.4	183.9	176.2	155.3	134.7	127.3	108.2
Changing rate	87.5 %	119.4 %	50.6 %	82.9 %	69.5 %	100.6 %	113.5 %	128.7 %	115.1 %	82.4 %	82.2 %	91.2 %	80.9 %

Source of the number of breast cancer deaths: “Vital Statistics”. Source of crude morbidity rate and mortality rate: Center for Cancer Control and Information

### Future projection of COI from 2014 to 2020 (fixed model)

Table 3 shows the future projection of COI based on a fixed model. COI was estimated to be 704.7 billion yen in 2014, 705.9 billion yen in 2017, and 703.4 billion yen in 2020; thereby increasing until 2017 and then decreasing in 2020. The rate of change from 2011 to 2020 was 0.9 %. The direct costs increased until 2020, whereas the morbidity costs decreased in 2017 and the mortality costs decreased from 2014. The rate of change for each component was stable during these 10 years at less than 5 %. Nonetheless, the mortality costs per person were projected to decrease continuously.

**Table 3:** Mortality rate and the number of deaths due to breast cancer (1996 and 2011)

Model	Item	2011	2014	2017	2020
	Estimated population (thousand person)	127,799	126,949	125,739	124,223
	[% of 65 years or older]	23.1 %	26.1 %	28.0 %	29.1 %
Fixed model	Number of breast cancer deaths (person)	12,791	13,308	13,641	13,901
	[% of 65 years or older]	52.1 %	56.2 %	58.7 %	59.4 %
	Average age of death (years)	66.1	66.8	67.4	67.9
	Direct cost (billion yen)	166.3	168.3	170.0	170.1
	Morbidity cost (billion yen)	46.8	48.6	48.7	48.4
	Mortality cost (billion yen)	484.0	487.8	487.2	484.8
	[% of 65 years or older]	23.1 %	25.6 %	27.0 %	26.7 %
	Mortality cost per person (million yen)	38.0	36.7	35.7	34.9
	COI (billion yen)	697.0	704.7	705.9	703.4
Linear model	Number of breast cancer deaths (person)	12,791	13,968	15,015	16,039
	[% of 65 years or older]	52.1 %	56.0 %	59.4 %	60.8 %
	Average age of death (years)	66.1	66.9	67.9	68.7
	Direct cost (billion yen)	166.3	157.4	158.3	160.6
	Morbidity cost (billion yen)	46.8	47.3	49.3	46.5
	Mortality cost (billion yen)	484.0	509.5	523.5	536.4
	[% of 65 years or older]	23.1 %	25.5 %	27.7 %	28.2 %
	Mortality cost per person (million yen)	38.0	36.5	34.9	33.4
	COI (billion yen)	697.0	714.1	731.1	743.6
Logarithm model	Number of breast cancer deaths (person)	12,791	13,153	13,720	14,222
	[% of 65 years or older]	52.1 %	54.6 %	57.4 %	58.5 %
	Average age of death (years)	66.1	66.4	67.1	67.8
	Direct cost (billion yen)	166.3	158.8	154.7	153.0
	Morbidity cost (billion yen)	46.8	45.8	44.3	44.0
	Mortality cost (billion yen)	484.0	494.6	499.5	502.4
	[% of 65 years or older]	23.1 %	24.2 %	25.7 %	25.7 %
	Mortality cost per person (million yen)	38.0	37.6	36.4	35.3
	COI (billion yen)	697.0	699.2	698.5	699.4
Mixed model	Number of breast cancer deaths (person)	12,791	13,555	14,323	14,986
	[% of 65 years or older]	52.1 %	54.2 %	56.8 %	57.4 %
	Average age of death (years)	66.1	66.3	67.0	67.5
	Direct cost (billion yen)	166.3	157.1	159.6	162.2
	Morbidity cost (billion yen)	46.8	45.5	46.5	47.3
	Mortality cost (billion yen)	484.0	510.9	524.0	534.3
	[% of 65 years or older]	23.1 %	24.4 %	26.1 %	25.9 %
	Mortality cost per person (million yen)	38.0	37.7	36.6	35.7
	COI (billion yen)	697.0	713.5	730.0	743.8

Source of estimated population: 2008; Ministry of Internal Affairs and Communications “Population Estimates” 2014 2020; National Institute of Population and Social Security Research “Population Statistics of Japan”

The fixed model assumes that health-related indicators were fixed at 2011 levels and only demographic changes had any impact on COI. According to the National Institute of Population and Social Security Research, the Japanese population began to decrease in 2008 and it was estimated to continue to decrease until 2020, while the rate of aging was estimated to rise [24]. Under these conditions, the number of deaths from breast cancer was estimated to increase by 8.7 % (APC of 0.9 %) from 2011 to 2020. The number of deaths in persons aged 65 years or older was predicted to increase by 7.4 % and the average age at death was also predicted to rise by 1.8 years. In the fixed model, the mortality rate of 2014, 2017 and 2020 was fixed at 2011 level, and it was considered that increase of aged population had impact on such aging of persons who died by breast cancer.

### Future projection of COI from 2014 to 2020 (variable model)

**Fig. 1:**
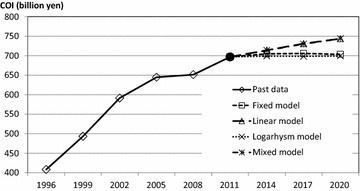
The trends of cost of illness (COI) by prediction models

Using a linear model, COI in 2014, 2017, and 2020 was estimated to be 714.1 billion yen, 731.1 billion yen, and 743.6 billion yen, respectively. Using a logarithmic model the predictions were valued at 699.2 billion yen, 698.5 billion yen, and 699.4 billion yen, respectively, and lastly, using a mixed model, COI in 2014, 2017, and 2020 was estimated to be 713.5 billion yen, 730.0 billion yen, and 743.8 billion yen, respectively. Figure 1 shows the trends of COI based on the fixed model as well as the 3 variable models.

**Fig. 2:**
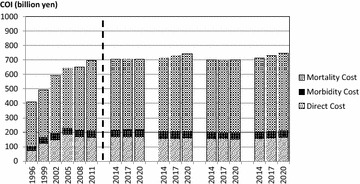
The cost of illness (COI) projections with cost elements

Since the trend of each health related indicator was different, the monotype estimation (logarithmic model or linear model) might not predict future COI precisely. The mixed model was a combination of models of higher coefficient of determination and, therefore, considered the most valid model in this study. According to this mixed model, COI showed a 6.7 % (APC: 0.7 %) increase from 2011 to 2020. The direct and morbidity costs were stable, but the mortality costs increased 10.4 % (APC: 1.1 %). As the number of deaths increased by 17.2 % (APC: 1.8 %), the mortality costs per person decreased continuously. Figure 2 shows the trends of each COI component in each model.

### Comparison with COI of stomach cancer

Our previous study of COI of stomach cancer estimated COI in 1996, 2002, 2008, 2014, and 2020 using the year 2008 as the benchmark. In that study, COI was estimated to be 1114.2 billion yen (direct costs: 253.7 billion yen, morbidity costs: 54.0 billion yen, and mortality costs: 806.4 billion yen) in 2008 and was predicted to decrease to 484.5 billion yen in 2020 using a mixed model (direct costs: 100.4 billion yen, morbidity costs: 26.4 billion yen, and mortality costs: 357.7 billion yen). The decrease in COI from 2008 to 2020 was 56.5 % (APC: 8.8 %), and the mortality cost was also predicted to decrease by 55.6 % (APC: 8.6). However, there was a large variation in COI estimation (70.1 % variation to COI in 2008) by each method [12].

Compared with COI of stomach cancer, COI of breast cancer was smaller in the benchmark year but was predicted to increase continuously to be 1.5-times that of stomach cancer by 2020. The mortality costs of breast cancer were estimated to be smaller, but were predicted to be 1.5-times those of stomach cancer. Moreover, there was less variation in the estimation of COI of breast cancer, which was only 6.4 % of the benchmark year’s COI. Each estimation method showed that COI of breast cancer was stable or increased modestly.

## Discussion

The results of this study demonstrated that COI of breast cancer increased significantly from 1996 to 2011. The increase in the mortality costs contributed significantly. Furthermore, it was predicted that COI would continue to trend upwards until 2020, but the rate of increase would decline. The annual average rate of increase was 3.8 % from 1996 to 2011 but was predicted to be only 0.7 % from 2011 to 2020 in the mixed model. Because the variation in the model was small, we can conclude that future COI will be stable or increase only slightly.

COI and the mortality costs of breast cancer are predicted to exceed those of stomach cancer in the near future. Changes in the number of deaths and the average age at death were the main causes of the increase in the mortality costs. Because the mortality costs were calculated as the number of deaths multiplied by the mortality costs per person (lifetime labor-value per person) according to sex and 5 age-groups, the increase in the number of deaths had a direct influence on the increase in the mortality costs. Additionally, the lifetime labor-value per person (or human capital value) differs according to the average age at death. Because the human capital value of the 25 to 29-age-group was the highest, an increase in the average age at death resulted in decreased mortality costs. Regarding the number of deaths from benchmark year to 2020, for stomach cancer, there was a 25.1 % decrease (APC: −2.4 %), and conversely, for breast cancer, there was a 17.2 % increase (APC: 1.8). Nonetheless, the number of deaths from stomach cancer was predicted to be 37,581, whereas that from breast cancer was predicted to be only 14,986. However, the mortality costs per person were predicted to be 9.5 million yen for stomach cancer and 35.7 million yen for breast cancer. This difference is most likely because of the difference in the average age at death, which was predicted to be 79.1 years for stomach cancer and 67.5 years (11.6 years younger) for breast cancer in 2020. Moreover, while the annual rate of increase in the average age at death from stomach cancer was stable before and after the benchmark year (0.5 %), the annual rate of increase in the average age at death from breast cancer declined after the benchmark year (from 0.6 to 0.2 %).

There are two types of cancers: one affects an “older group” (74.8 years old for stomach cancer, 74.6 years old for lung cancer, 75.6 years old for colon cancer, and so on in 2011) and the other affects a “younger group” (66.1 years old for breast cancer, 67.6 years old for cervical cancer, and so on in 2011) [14]. The trends of the mortality rates by age suggest that the rate of increase of the average age at death will probably rise for the older group and decline for the younger group. Accordingly, the mortality costs per person and COI will probably decrease for the older group and increase for the younger group. These results may be very useful for prioritizing policies for cancer control.

The effectiveness of mammography for the detection of breast cancer has already been proven by several studies [25, 26]. While the assessment of technology is outside the scope of this study, our results demonstrate the importance of allocating subsidies to such countermeasures preferentially to implement efficient policy.

There are some limitations to this study that complicate the interpretation of the data used for approximations. Firstly, the study period was relatively short and there were dramatic changes within the healthcare system during this time. However, the variation among the different methods for determining projections was small and, therefore, the projections are likely to be accurate for the near future. Additionally, we could not predict future increases in the labor-value per person, particularly with regard to the employment and compensation rates for women, which are likely to rise. When these are taken into account, COI of breast cancer is likely to increase even more, which would only further strengthen our conclusions.

## Conclusions

The findings of the present study suggest that COI of breast cancer has continuously increased up to the present and that trend is likely to continue, although the pace may be expected to decline. The average age at death from breast cancer was less than that from other cancers and the pace of aging was slow. These factors contribute to increasing the social burden of breast cancer, making it clear that policies to mitigate these effects are critical.

## Abbreviations

COI: cost of illness
APC: annual percent change
